# Preoperative Intestinal Preconditioning to Prevent Postoperative Vasoplegia After Tricuspid Valve Surgery

**DOI:** 10.1016/j.atssr.2025.11.008

**Published:** 2025-12-05

**Authors:** Paulina Ramirez-Jaramillo, Mateo Marin-Cuartas, Bettina Pfannmüller, Manuela De La Cuesta, Jagdip Kang, Zara Dietze, Suzanne De Waha, Madlen Uhlemann, Thilo Noack, David Holzhey, Alexey Dashkewich, Martin Misfeld, Michael A. Borger, Philipp Kiefer

**Affiliations:** 1 University Department of Cardiac Surgery, Leipzig Heart Center, Leipzig, Germany; 2 Department of Cardiac Surgery, Zurich University Hospital, Zurich, Switzerland; 3 Department of Electrophysiology, Schleswig-Holstein University Hospital, Lübeck Campus, Lübeck, Germany; 4 Department of Cardiothoracic Surgery, Royal Prince Alfred Hospital, Sydney, New South Wales, Australia; 5 Institute of Academic Surgery, Royal Prince Alfred Hospital, Sydney, New South Wales, Australia; 6 The Baird Institute of Applied Heart and Lung Surgical Research, Sydney, New South Wales Australia; 7 Sydney Medical School, University of Sydney, Sydney, New South Wales, Australia

## Abstract

**Background:**

Right ventricular dysfunction may lead to intestinal bacterial translocation secondary to portal venous hypertension, thereby contributing to systemic inflammatory response syndrome and vasoplegia after tricuspid valve surgery. This study aimed to evaluate the impact of preoperative intestinal preconditioning (oral antibiotics and laxatives) on the occurrence of postoperative vasoplegia after tricuspid valve surgery.

**Methods:**

This retrospective single-center analysis included patients who underwent tricuspid valve surgery from 2017 to 2021. The outcomes of patients who received preoperative intestinal preconditioning were compared with the outcomes of patients who did not. Primary outcomes were in-hospital mortality and the occurrence of postoperative vasoplegia. Secondary outcomes were intensive care unit (ICU) and hospital length of stay (LOS).

**Results:**

Among 142 patients, 44 (30.9%) received preoperative preconditioning, whereas 98 (69.0%) did not. Baseline characteristics were similar between the groups. In-hospital mortality rates were comparable (6.8% vs 5.1%, with and without preconditioning, respectively; *P* = .7). There was no difference in the mean hospital LOS (21 [12] days with preconditioning vs 18 [11] days without; *P* = .4). Vasoplegia occurred in 0 (0%) patients with intestinal preconditioning vs 9 (9.2%) patients without preconditioning (*P* = .04). In a subgroup analysis, patients without vasoplegia had significantly lower mortality (3.7% vs 33.3%; *P* = .001), shorter mean ICU LOSs (3 [6] days vs 12 [20] days; *P* = .010) and hospital LOS (19 [13] days vs 23 [18] days; *P* = .134).

**Conclusions:**

Preoperative intestinal preconditioning before tricuspid valve surgery may prevent postoperative vasoplegia. Lower vasoplegia rates are associated with reduced ICU and hospital LOSs and improved in-hospital mortality.

## Visual Abstract

**Figure:**
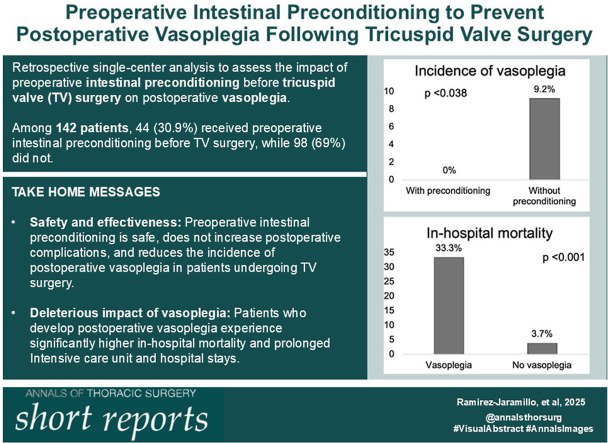

**In Short**

▪ Preoperative intestinal preconditioning is safe, does not increase postoperative complications, and reduces the incidence of postoperative vasoplegia in patients undergoing TV surgery.
▪ Patients who experience postoperative vasoplegia have significantly higher in-hospital mortality and prolonged ICU and hospital LOS. A prospective study with a sufficient sample size is warranted to validate the impact of preoperative intestinal preconditioning on clinical outcomes.

Significant tricuspid regurgitation can lead to right ventricular dysfunction and retrograde venous congestion. (^1^Portal hypertension caused by venous stasis results in local hypoxia, which damages the intestinal mucosa through oxidative stress. This damage impairs intestinal permeability and delays intestinal transit.^1^ Stagnant luminal contents combined with a devitalized bowel wall promote bacterial overgrowth, playing a crucial role in bacterial translocation.^1^^,^^2^ Bacteria and endotoxins trigger local immune activation and cytokine production. This process results in increased vessel permeability, which facilitates the passage of bacteria from the intestines to extraintestinal sites.^1^^,^^2^ This bacterial translocation is closely linked to systemic inflammatory response syndrome and resulting vasoplegia.^2^, ^3^, ^4^ Prophylactic intestinal preconditioning with oral antibiotics and laxatives may reduce bacterial load and improve intestinal transit, thus potentially decreasing bacterial translocation and vasoplegia.^5^^,^^6^

This study evaluated the effect of preoperative intestinal preconditioning on early outcomes after tricuspid valve (TV) surgery. We hypothesized that a protocol of oral antibiotics and laxatives 24 hours preoperatively would reduce postoperative vasoplegia incidence and improve clinical outcomes.

## Material and Methods

### Ethical Statement

The study was approved by the Ethics Committee of the University of Leipzig (136/22-ek; March 17, 2023). Because of the study’s retrospective design, individual patient informed consent was waived.

### Study Cohort

This study included patients who underwent elective TV surgery between 2017 and 2021 at our institution (University Department of Cardiac Surgery, Leipzig Heart Center, Leipzig, Germany), grouped by whether preoperative intestinal preconditioning was performed. The decision was made at the surgeon’s discretion. Patients with cardiogenic or septic shock, mechanical circulatory support, emergency procedures, infective endocarditis, aortic surgery, or ventricular assist device implantation were excluded. Preoperative characteristics, intraoperative data, and postoperative outcomes were collected from a computerized database, with additional study-specific variables obtained from patients’ charts.

### Study Outcomes

The primary outcomes were in-hospital mortality and the occurrence of postoperative vasoplegia. Secondary outcomes included intensive care unit (ICU) and hospital length of stay (LOS). Postoperative vasoplegia was defined as hypotension (mean arterial pressure <60 mm Hg), oliguria (<0,5 mL/kg/h), and a high requirement for vasopressors (norepinephrine >0.5 μg/kg/min or use of terlipressin, or both).^7^

### Prophylactic Preoperative Intestinal Preconditioning

The preconditioning protocol included oral antibiotics and laxatives: 550 mg rifaximin orally once daily for 24 hours preoperatively, 800 mL tea or water with 100 mg maltodextrin, fiber powder, and a lactulose enema.

### Statistical Analysis

Categorical variables were analyzed using the χ^2^ test, and continuous variables were analyzed using the Wilcoxon-Mann-Whitney test, depending on the normality of the data. Continuous variables were presented as mean (SD), and categorical variables were presented as counts and percentages. A *P* value of <.05 was considered statistically significant. All tests were performed using R software version 2024.04.1+748 (R Foundation for Statistical Computing).

## Results

### Baseline Demographics and Intraoperative Data

A total of 142 patients were analyzed: 44 (30.9%) received preoperative intestinal preconditioning, and 98 (69%) did not receive it. Baseline characteristics were comparable between groups with the exception of age. Patients who underwent intestinal preconditioning were significantly younger than control patients (68 [11] years vs 73 [10]; *P* = .011). There was no significant difference between the 2 groups in terms of right ventricular variables, including right ventricular end-diastolic diameter, tricuspid annular plane systolic excursion, and systolic pulmonary artery pressure. Intraoperative variables, including cardiopulmonary bypass and cross-clamp times, were similar between both groups (Tables 1, 2). However, patients who had intestinal preconditioning were more likely to undergo on-pump beating heart TV surgery (29.5% vs 6.1%; *P* < .001).

**Table 1:** Baseline Characteristics

Characteristics	Preconditioning n = 44	No Preconditioning n = 98	*P* Value
Age, y	68 [11]	73 [10]	.011
BMI, kg/m^2^	27.2 [5.4]	27.5 [5.6]	.356
Male sex	20 (45.4)	42 (42.8)	.773
Atrial fibrillation	28 (63.6)	66 (67.3)	.268
Diabetes mellitus	9 (20.4)	25 (25.5)	.514
COPD	4 (9.1)	9 (9.2)	.986
Peripheral vascular disease	8 (18.2)	12 (12.2)	.347
Stroke	13 (29.5)	4 (4.1)	.479
Myocardial infarction	13 (29.5)	4 (4.1)	.479
Dialysis	0 (0)	1 (1.0)	.501
LVEF, %	56 [9]	54 [9]	.298
Previous cardiac surgery	19 (43.2)	37 (37.7)	.541
RVEDD, mm	44 [11]	41 [12]	.496
TAPSE, mm	16 [5]	16 [6]	.327
sPAP, mm Hg	42 [29]	44 [21]	.604

Categorical variables are expressed as number (percentage); continuous variables are expressed as mean [SD].

BMI, body mass index; COPD, chronic obstructive pulmonary disease; LVEF, left ventricular ejection fraction; RVEDD, right ventricular end-diastolic diameter; sPAP, systolic pulmonary artery pressure; TAPSE, tricuspid annular plane systolic excursion.

**Table 2:** Intraoperative Details and Early Postoperative Outcomes

Intraoperative Details and Outcomes	Preconditioning n = 44	No Preconditioning n = 98	*P* Value
Intraoperative details			
Isolated TV replacement	17 (38.6)	31 (31.6)	.415
Isolated TV repair	27 (61.4)	68 (69.4)	.347
Concomitant procedures			
Mitral valve surgery	24 (54.5)	63 (64.3)	.271
LAA closure	8 (18.2)	13 (13.3)	.445
PFO/ASD closure	4 (9.1)	6 (6.1)	.523
Atrial fibrillation ablation	10 (22.7)	19 (19.4)	.648
CPB time, min	113 [69]	114 [40]	.874
Cross-clamp time, min	53 [63]	62 [38]	.284
On-pump beating heart surgery	13 (29.5)	6 (6.1)	<.001
Postoperative outcomes			
Reexploration for bleeding	5 (11.4)	16 (16.3)	.441
Acute kidney injury requiring dialysis	10 (22.7)	22 (22.4)	.971
Cardiogenic shock	12 (27.3)	22 (22.4)	.533
ECMO	1 (2.3)	3 (3.1)	.793
Stroke	3 (6.8)	4 (4.1)	.486
New-onset atrial fibrillation	30 (68.2)	57 (58.2)	.416
Multiorgan dysfunction syndrome	2 (4.5)	5 (5.1)	.887
Vasoplegia	0 (0)	(9.2)	.038
In-hospital mortality	3 (6.8)	5 (5.1)	.682
ICU length of stay, d	3 [6]	4 [5]	.530
Hospital length of stay, d	21 [12]	18 [11]	.453

Categorical variables are expressed as number (percentage); continuous variables are expressed as mean [SD].

ASD, atrial septal defect; CPB, cardiopulmonary bypass; ECMO, extracorporeal membrane oxygenation; ICU, intensive care unit; LAA, left atrial appendage; PFO, permanent foramen ovale; TV, tricuspid valve.

### Early Postoperative Outcomes

Early postoperative outcomes, including complications such as major bleeding, acute kidney injury, and cardiogenic shock, showed no significant differences between groups (Table 2). In-hospital mortality was also comparable between the groups (6.8% vs 5.1%; *P* = .7). However, vasoplegia was not observed in the preconditioning group (0%), although it occurred in 9 patients (9.2%) in the no preconditioning group (*P* = .038). The mean ICU LOS was similar in both groups (6 [6] days vs 4 [5] days [*P* = .5], in the preconditioning and no preconditioning groups, respectively), and the mean hospital LOS was also similar in both groups (21 [12] days vs 18 [11] days [*P* = .4], in the preconditioning and no preconditioning groups, respectively).

A subgroup analysis revealed significantly lower in-hospital mortality rates (3.7% vs 33.3%; *P* < .001) and shorter mean ICU LOS (3 [6] days vs 12 [20] days; *P* = .010) among patients who did not experience vasoplegia compared with patients who did. Mean hospital LOS was also shorter in patients without vasoplegia (19 [13] days vs 23 [18] days; *P* = .134), although no statistical significance was reached (Table 3, Figure).

**Table 3:** Subanalysis: Comparison of Main Study Outcomes in Patients With and Without Postoperative Vasoplegia

Outcomes	Vasoplegia n = 9	No Vasoplegia n = 133	P Value
In-hospital mortality	3 (33.3)	5 (3.7)	<.001
ICU length of stay, d	12 [20]	3 [6]	.010
Hospital length of stay, d	23 [18]	19 [13]	.134

Categorical variables are expressed as number (percentage); continuous variables are expressed as median [SD].

ICU, intensive care unit.

**Figure.**
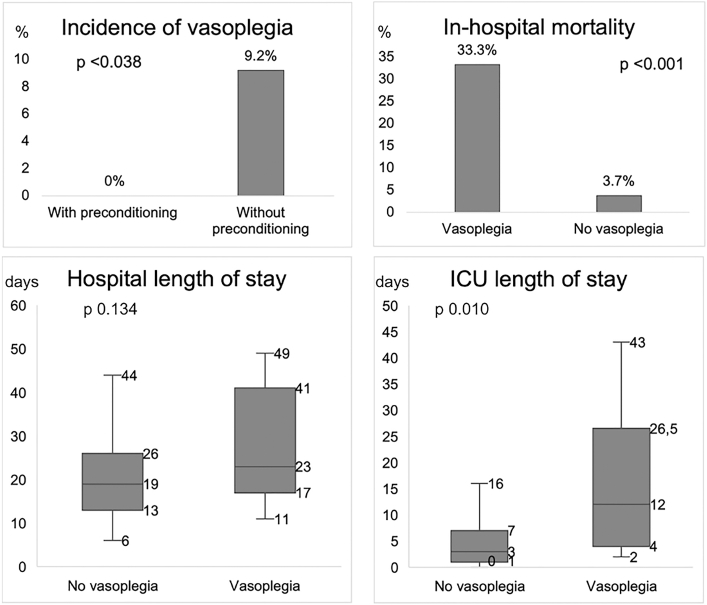
Summary of main study outcomes. (ICU, intensive care unit.)

## Comment

This study evaluated the impact of preoperative intestinal preconditioning in patients undergoing elective TV surgery. The main results were as follows:

• Safety and effectiveness: Preoperative intestinal preconditioning is safe, it does not increase postoperative complications, and it reduces the incidence of postoperative vasoplegia in patients undergoing TV surgery.
• Deleterious impact of vasoplegia: Patients who experience postoperative vasoplegia have significantly higher in-hospital mortality and prolonged ICU and hospital LOS. A prospective study with a sufficient sample size is warranted to validate the impact of preoperative intestinal preconditioning on clinical outcomes.

Our study suggests that the use of preoperative intestinal preconditioning could prevent vasoplegia in patients undergoing TV surgery.

### Vasoplegia in Cardiac Surgery

Vasodilatory shock is a frequent complication in cardiovascular surgery; it affects between 5% and 25% of patients, with higher rates (up to 50%) in patients with known risk factors.^8^ Although many cases resolve with treatment, severe vasoplegia is associated with substantial morbidity and mortality. The precise triggers for postsurgical vasoplegia remain unclear.^9^

One potential mechanism is gut dysbiosis, characterized by the loss of beneficial microbes and the overgrowth of pathogenic bacteria. Dysbiosis is strongly associated with worse clinical outcomes, including higher infection rates, mortality, and prolonged hospital LOS.^2^ In patients with severe tricuspid regurgitation, portal hypertension leads to intestinal congestion and bacterial translocation, which may contribute to systemic inflammation and vasoplegia.

A study by Salameh and colleagues^10^ identified gut dysbiosis as an independent risk factor for increased mortality in critically ill patients. These investigators introduced the Microbiome Mortality Index and showed that patients with a high index value had a significantly higher 28-day mortality risk (hazard ratio, 2.2; 95% CI, 1.1-4.3). This finding supports our observation that intestinal preconditioning reduces the incidence of postoperative vasoplegia, which is a clinical surrogate of the degree of bacterial translocation and is strongly related to early postoperative mortality.

### Gut Bacteria and Cardiovascular Outcomes

There is growing evidence that intestinal bacterial translocation into the bloodstream, especially resulting from intestinal hypoperfusion and increased permeability after myocardial infarction, contributes to future cardiovascular events by triggering inflammation. Some studies suggest that preventing gut microbial translocation can protect cardiomyocytes and reduce cardiac mortality.^4^ For instance, the STAMINA trial (South Thames Trial of Antibiotics in Myocardial Infarction and unstable Angina)^8^ demonstrated that patients who had experienced myocardial infarction and who were treated with antibiotics had a 36% reduction in cardiac death and readmission for acute coronary syndrome over a 1-year follow-up period. This finding aligns with those of the present study, where reducing intestinal bacterial load and permeability through preoperative intestinal preconditioning lowered the incidence of vasoplegia, a key determinant of postoperative mortality.

### Cost-Effectiveness of Preoperative Intestinal Preconditioning

Preoperative intestinal preconditioning is a simple, noninvasive, and low-cost intervention. If effective in reducing vasoplegia, ICU LOS, and overall hospitalization, it could enhance patient outcomes and reduce health care costs. Despite promising results, further evidence is needed to confirm its clinical impact. A randomized clinical trial comparing intestinal preconditioning with standard care in patients undergoing TV surgery is particularly warranted.

### Study limitations

Given that this was an observational study, procedural selection bias is a limitation. The use of preoperative intestinal preconditioning was left to the surgeon’s discretion because it is not mandatory in our center. The lack of outpatient follow-up limits conclusions beyond the hospital stay. However, we believe that perioperative vasoplegia likely affects short-term rather than long-term outcomes. Additional prospective studies will be necessary to assess the effects and potential benefits of this preventive approach further.

### Conclusion

Preoperative intestinal preconditioning is a safe strategy for patients undergoing TV surgery, it does not increase early postoperative complications, and it may prevent postoperative vasoplegia. Lower vasoplegia rates could reduce ICU and hospital LOS and improve in-hospital mortality.
